# Improved Network and Training Scheme for Cross-Trial Surface Electromyography (sEMG)-Based Gesture Recognition

**DOI:** 10.3390/bioengineering10091101

**Published:** 2023-09-20

**Authors:** Qingfeng Dai, Yongkang Wong, Mohan Kankanhali, Xiangdong Li, Weidong Geng

**Affiliations:** 1 College of Computer Science and Technology, Faculty of Computer, Zhejiang University, Hangzhou 310058, China; qfdai@zju.edu.cn (Q.D.); axli@zju.edu.cn (X.L.); 2 School of Computing, National University of Singapore, 21 Lower Kent Ridge Rd, Singapore 119077, Singapore; yongkang.wong@nus.edu.sg (Y.W.); mohan@comp.nus.edu.sg (M.K.); 3 Zhejiang Lab, Hangzhou 310058, China

**Keywords:** surface electrography (sEMG), deep learning, gesture recognition, mutual information, knowledge distillation

## Abstract

To enhance the performance of surface electromyography (sEMG)-based gesture recognition, we propose a novel network-agnostic two-stage training scheme, called *sEMGPoseMIM*, that produces trial-invariant representations to be aligned with corresponding hand movements via cross-modal knowledge distillation. In the first stage, an sEMG encoder is trained via cross-trial mutual information maximization using the sEMG sequences sampled from the same time step but different trials in a contrastive learning manner. In the second stage, the learned sEMG encoder is fine-tuned with the supervision of gesture and hand movements in a knowledge-distillation manner. In addition, we propose a novel network called *sEMGXCM* as the sEMG encoder. Comprehensive experiments on seven sparse multichannel sEMG databases are conducted to demonstrate the effectiveness of the training scheme *sEMGPoseMIM* and the network *sEMGXCM*, which achieves an average improvement of +1.3% on the sparse multichannel sEMG databases compared to the existing methods. Furthermore, the comparison between training *sEMGXCM* and other existing networks from scratch shows that *sEMGXCM* outperforms the others by an average of +1.5%.

## 1. Introduction

In human–computer interfaces (HCIs), hand movements commonly offer a natural way for users to interact with the computer [1]. There are multiple ways to recognize hand gestures, such as vision- [2], WiFi- [3], and radar-based approaches with off-body sensors [4], as well as approaches based on biosignals such as surface electromyography (sEMG) and electroencephalography (EEG) [5,6]. Among these approaches, the sEMG-based muscle–computer interface is attracting increasing attention due to its robustness to the deployment environment and its non-invasive nature [7].

**Figure 1:**
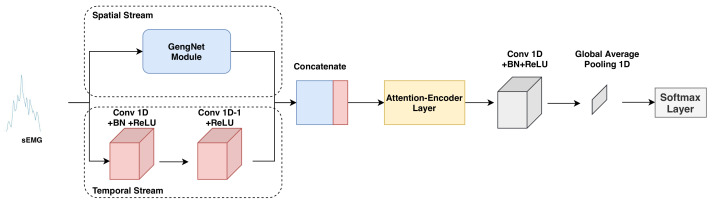
The architecture of *sEMGXCM*, which is end-to-end and double-stream, used as the backbone of $E_{semg}$.

With the recent advancement of deep learning techniques, a common method for sEMG-based gesture recognition is to translate the sEMG signals to images and then Convolutional Neural Network (CNN) [5,8,9] is trained for classification. However, these models only capture the spatial information of sEMG signals without considering the temporal information. To address this issue, recurrent neural networks (RNNs) [10] and the hybrid CNN–RNN [11,12] are adopted to extract both spatial and temporal features from sEMG signals and achieve better performances compared to CNN. However, RNN and CNN–RNN are rarely used in real-time HCIs due to their slow computation. Motivated by this fact, we propose an improved network, namely *sEMGXCM* (Figure 1). In this network, spatial and temporal features are extracted in parallel using 2D and 1D convolutional layers, respectively. After the extracted features are fused, a self-attention layer [13] is added to model the association across electrodes. To validate the effectiveness of *sEMGXCM*, we conducted a fair comparison between *sEMGXCM* and other three existing deep networks, GengNet [5], XceptionTime [9], and XCM [14]. The performances of these networks are obtained by training them from scratch and adopting cross-trial gesture recognition accuracy as the evaluation metric.

**Figure 2:**
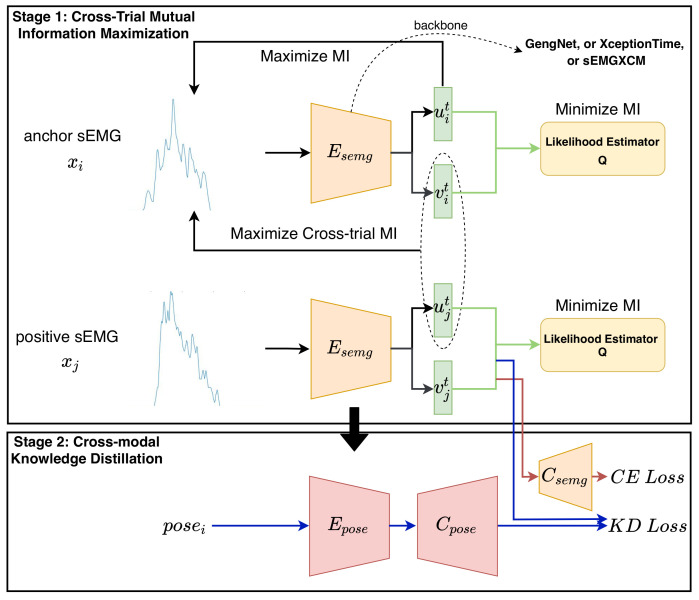
An overview of our proposed network-agnostic training framework, namely *sEMGPoseMIM*, for intra-subject sEMG-based gesture recognition. The positive sEMG $x_{j}$ is sampled from a different trial from that of the anchor sEMG $x_{i}$ at the same time window.

Despite the improvement brought by network design, cross-trial gesture-recognition performance is still far from optimal. A trial commonly represents a repetition of performing a hand gesture when wearing electrodes without removing them [15]. Then, the cross-trial gesture recognition accuracy could indicate the performance of a trained classification model during the longtime use of an sEMG-based application. Thus, it is essential to build a classification model with high cross-trial gesture-recognition accuracies. Motivated by the cross-modal association between sEMG signals and hand movements [16], we aim to model another type of association across different trials within the same sEMG modality. Based on these two kinds of associations, we propose a novel scheme, called *sEMGPoseMIM* (Figure 2), to enhance the training of sEMG-based classification models, such as GengNet [5], XceptionTime [9], and XCM [14].

Specifically, *sEMGPoseMIM* consists of two stages that, respectively, model the cross-modal (i.e., sEMG signals and hand movements) association and invariant information across different trials. In the first stage, inspired by the study of mutual information (MI) [17], we aim to train an encoder that generates trial-invariant representations. To do this, we sample pairs of sEMG sequences from different trials in the same time step. Then, the sEMG sequences of a pair are fed into the encoder, whose output is disentangled into a gesture-relevant representation and a trial-relevant representation. Subsequently, the mutual information between the two representations from a single sEMG sequence is minimized through a likelihood estimator to ensure the disentanglement, as Belghazi et al. do in [18]. In addition, the cross-trial mutual information between the gesture-relevant representation and trial-relevant representation from the two respective sEMG sequences of a sampled pair is maximized to mitigate the impact across different trials. In this way, an encoder producing trial-invariant representations is obtained. In the second stage, we aim to leverage the invariance of hand movements across different trials. To this end, we adopt a common knowledge-distillation method [19] to align the feature spaces of two modalities (i.e., sEMG signals and hand movements). Firstly, a teacher network of the hand movements modality is supervisedly trained to classify hand gestures. Next, a student network based on sEMG signals is initialized using the parameters learned in the first stage and then is jointly trained through classification loss as well as Kullback–Leibler divergence loss to the output of the well-trained teacher network. We validate the effectiveness of *sEMGPoseMIM* by comparing the performance of training using the scheme *sEMGPoseMIM* with that of training from scratch. In addition, the effect of the components of *sEMGPoseMIM* is verified.

The main contributions of this paper are summarised as follows.

- We design a new end-to-end convolutional neural network for cross-trial sEMG-based gesture recognition, namely *sEMGXCM*, that captures the spatial and temporal features of sEMG signals as well as the association across different electrodes. The parameter number of the self-attention layer increases as the number of electrodes increases, so *sEMGXCM* is utilized for sparse multichannel sEMG signals.
- We present a novel two-stage training scheme called *sEMGPoseMIM* for cross-trial sEMG-based gesture recognition. Specifically, the first stage is designed to maximize the mutual information between the pairs of cross-trial features at the same time step to produce trial-invariant representations. And the second stage models the cross-modal association between sEMG signals and hand movements via cross-modal knowledge distillation to enhance the performance of the trained network.
- A comprehensive evaluation of the proposed network *sEMGXCM* on the benchmark NinaPro databases is conducted, and the results show the superiority of *sEMGXCM* for cross-trial gesture recognition. Specifically, compared with the state-of-the-art network, *sEMGXCM* achieves improvements of +0.7%, +1.3%, +0.5%, +0.3%, +0.3%, +0.6%, and +1.0% on NinaPro DB1-DB7 [20,21,22,23]. We also performed an evaluation of our training scheme *sEMGPoseMIM* on NinaPro DB1-DB7. The experimental results show the superiority of *sEMGPoseMIM* for enhancing the cross-trial gesture recognition performance of networks. And the recognition accuracy of *sEMGXCM* from training it using *sEMGPoseMIM* is significantly higher than the state-of-the-art method by +1.3%, +1.5%, +0.8%, +2.6%, +1.7%, +0.8% and +0.6% on NinaPro DB1-DB7.

## 2. Related Work

### 2.1. sEMG-Based Gesture Recognition

The sEMG signal is recorded using electrode contact with the skin during the contraction of skeletal muscles [24], which is non-invasive and robust to environmental conditions. Recently, sEMG-based gesture recognition has attracted much attention due to its broad potential in the area of sign language, medical rehabilitation, virtual reality, and so on [25]. The approaches to tackle this classification problem can be categorized into conventional machine-learning-based approaches and deep-learning-based ones [5,7,9,11,26]. The former usually consists of three steps, including preprocessing sEMG signals, handcrafted feature extraction, and classification using the extracted features. Various handcrafted sEMG features are adopted, such as temporal–spatial descriptors (TSDs) [27], Discrete Wavelet Transform Coefficients (DWTCs) [28], and Continuous Wavelet Transform Coefficients (CWTCs) [29]. Given the extracted features, the conventional machine learning classifiers, such as the Support Vector Machine (SVMs) [30] and Random Forests [23], are employed for classification. However, handcrafted feature extraction often requires domain expertise and the manual engineering of features, which can be time-consuming and resource-intensive. In contrast, deep models could automatically learn relevant features from raw data, eliminating the need for explicit feature engineering. For example, Geng et al. [5] converts the sEMG signal into a grayscale image, and a network composed of multiple 2D convolutional layers is utilized to recognize it. However, 2D convolutional layers are hardly used to capture the temporal information of signals. Motivated by this fact, RNN [10] is specifically designed to handle sequential data, making it suitable for capturing temporal dependencies. Unlike traditional feed-forward networks, it possesses an internal memory that retains information about prior inputs. This memory enables RNNs to process data sequentially and consider the context of previous inputs when making predictions. Furthermore, the hybrid CNN–RNN [11] is proposed by combining the strengths of both CNNs and RNNs. CNNs excel at extracting spatial features through the use of convolutional filters, while RNNs specialize in handling sequential information. By integrating these two architectures, the hybrid model can simultaneously capture spatial and temporal features. Furthermore, XceptionTime [9] utilizes 1D convolutional layers to extract fine-grained temporal information in time-series sEMG data.

Besides the input of converted images, Côté-Allard et al. [31] employs spectrograms extracted from sEMG signals as the input of a CNN. Wei et al. [8] fed vectors of multiple handcrafted features into a multi-stream convolutional neural network, and the approach made significant improvements in cross-trial gesture recognition. During the collection of sEMG signals, data of other modalities may be collected simultaneously [20,21,22]. Therefore, multimodal gesture-recognition methods that fuse the features of multimodal data are introduced to achieve further improvement [32,33]. In contrast, Hu et al. [16] utilized the hand poses to model the cross-modal association via adversarial learning during the training phase and improved the cross-trial gesture recognition performance during the test phase, barely using sEMG signals. Our training scheme *sEMGPoseMIM* is also formulated as multimodal training but is a unimodal evaluation.

### 2.2. Mutual Information and Cross-Modal Learning

In this work, our target is to learn trial-invariant representations of sEMG signals and make use of multimodal data during the training phase. Recently, mutual information (MI) [34] has been widely used in representation learning such as subject-invariant brain–computer-interface [35] and view-invariant human-pose estimation [36]. However, for the applications of muscle–computer interfaces (MCIs), mutual information is often utilized to select channels [37] or features [38]. Unlike these approaches, the maximization of mutual information is used for trial-invariant representation learning in our work.

To model the inherent relationship between the sEMG signals and finger movements, cross-modal learning-based methods are reviewed next. We shall focus on the approaches designed for pattern recognition. Hu et al. [16] performed the cross-modal transformation of sEMG signals and hand movements to obtain a fused feature of these two modalities. Liu et al. [39] also utilized cross-modal transformation to obtain more discriminative imagined visual features from a single modality. In addition to cross-modal transformation, Gu et al. [40] mapped noisy data from RGBD and wearable sensors to accurate 4D representations of lower limbs to perform abnormal gait-pattern recognition via cross-modal transfer. Considering that finger movements are more discriminative and generalized for gesture recognition, we follow [41] to utilize cross-modal knowledge distillation instead of transformation to model the relationship between these two modalities.

## 3. Materials and Methods

### 3.1. sEMGXCM

In this subsection, we present our improved network for cross-trial sEMG-based gesture recognition (*sEMGXCM*). Specifically, we demonstrate the architecture of the network and then explain the novelty of *sEMGXCM*.

Existing networks for sEMG-based gesture recognition tend to only extract temporal features (e.g., XceptionTime [9]) or spatial features (e.g., GengNet [5]). The spatial information of sEMG signals could indicate the spatial arrangement of electrodes, such as ring-like and matrix-like arrangements, as well as the muscle activities of different muscle groups. On the other hand, temporal information could provide valuable insights into the dynamic nature of sEMG signals, and the temporal relationships between different signal segments allow for a more comprehensive understanding of the underlying physiological processes. These factors will lead to a more accurate classification of different hand gestures. Although handcrafted features can be extracted to cover both spatial and temporal scenarios [8], it is time-consuming to obtain them. Therefore, we follow the dual-stream architecture of XCM [14], which is designed for multivariate time series data classification, to simultaneously extract spatial and temporal features.

**Figure 3:**
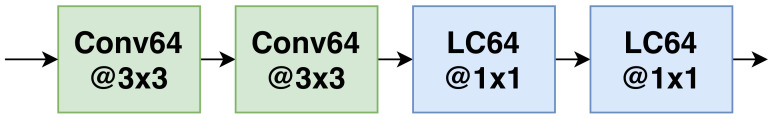
The architecture of the GengNet Module in the network *sEMGXCM*. Conv and LC, respectively, denote the 2D convolutional layer and 2D locally connected layer. The number following the layer name and the number after the ampersand denote the number of filters and the convolutional kernel size, respectively.

As shown in Figure 1, the temporal stream consists of two 1D convolution blocks, and the spatial one contains two 2D convolution layers and two 2D locally connected layers. The kernel size of the 1D convolution filters is set to W×C, where *W* and *C* denote the time window size and the number of electrodes, respectively. As 1D convolution filters slide over the time axis, the temporal stream shall capture the information across different timestamps. On the other hand, the spatial stream follows the architecture of GengNet [5] as shown in Figure 3. The locally connected layers of the spatial stream extract features of which electrodes indicate the specific hand gesture. Note that the hand movements are driven by specific muscle groups, and the features extracted by the spatial stream are explainable. Given the temporal and spatial features, a fusion operation is conducted, followed by a self-attention layer to learn the influence of different electrodes or time steps on gesture recognition. Specifically, inspired by [13], a four-head self-attention layer followed by a feedforward layer was adopted to not just focus on the current electrode or time step but also obtain information about the context. In the following step, we added the same aforementioned 1D convolution block and a 1D global average pooling to improve the generalization ability of *sEMGXCM*. Finally, we performed classification with a softmax layer.

In the field of image classification, 2D convolutional layers that apply multiple filters, each with different weights, can learn to extract different types of spatial information from images [42]. On the other hand, 1D convolutional layers are mainly used to extract temporal information from time-series signals, such as audio and speech [43,44]. In our network *sEMGXCM*, two streams that use 2D and 1D convolutional layers, respectively, to extract spatial and temporal features. In contrast, GengNet only uses 2D convolutional layers and XceptionTime only for 1D convolutional layers. Therefore, *sEMGXCM* can make use of both spatial and temporal information from the input sEMG signals. In addition to XCM [14], a self-attention layer is added to learn the information across different electrodes or time steps.

### 3.2. sEMGPoseMIM

In this subsection, we present a novel two-stage training scheme to enhance the networks for sEMG-based gesture recognition. As an instance of hybridization engineering [45], this training scheme (called *sEMGPoseMIM*, shown in Figure 2) is inspired by mutual information across different trials, as well as the inherent relationship between sEMG signals and hand movements. Specifically, we aim to generate trial-invariant representations from sEMG signals via maximizing cross-trial mutual information in the first stage. In the second stage, the initialized model is fine-tuned via cross-modal learning with another modality (i.e., hand movements).

In this work, mutual information maximization is applied during the training phase to learn a trial-invariant representation, which is significantly different from previous works, in which mutual information is used for channel or feature selection [46,47]. In addition, cross-modal knowledge distillation is utilized to capture the inherent correlation between sEMG signals and hand movements, enhancing the learned trial-invariant representation.

#### 3.2.1. Stage 1: Cross-Trial Mutual Information Maximization

Given an input sEMG sequence *x*, we aim to learn an encoder $E_{semg}$ to produce a trial-relevant representation *v* and a gesture-relevant representation *u*, while *v* and *u* are expected to be disentangled. In other words, two conditional distributions p(v|x) and p(u|x) are estimated by training the encoder $E_{semg}$. Therefore, we can recognize the same gesture of one subject from different trials.

An anchor sEMG sequence $x_{i}^{t}$ is constructed by capturing sEMG signals starting from time step *t* of the *i*-th trial. For each anchor sEMG sequence, we match a positive sEMG sequence $x_{j}^{t}$ that is sampled from the same time step of another trial *j*. Then, an encoder $E_{semg}$ is employed to generate a trial-relevant representation $v_{i}^{t}\in R^{d}$ and a gesture-relevant representation $u_{j}^{t}\in R^{d}$ given an sEMG sequence $x_{i}^{t}$. To learn a cross-trial representation, $E_{semg}$ is trained via the maximization of cross-trial mutual information using the following objective Equation (1):

(1) $$\max\left[\sum_{i}\underset{\mathrm{MI}}{\underset{︸}{I(x_{i}^{t};v_{i}^{t},u_{i}^{t})}}+\sum_{i\neq j}\underset{\mathrm{cross}-\mathrm{trial}\;\mathrm{MI}}{\underset{︸}{I(x_{i}^{t};v_{i}^{t},u_{j}^{t})}}\right]$$

where the first term is a conventional MI-based representation objective, and the second term maximizes the MI between the input sEMG sequence and its cross-trial counterpart. In this way, the learned representation, i.e., $\left(v_{i}^{t},u_{i}^{t}\right)$, could capture the gesture-relevant information maintained from different trials.

In fact, the gesture-relevant representation *u* and trial-relevant representation *v* are conditionally independent as they are assumed to be disentangled. To ensure this disentanglement between *u* and *v*, a regularization term $L_{\mathrm{inter}}$ based on their mutual information is introduced. The information for *u* and *v* shall be made mutually exclusive by minimizing this regularization term $L_{\mathrm{inter}}$.

Considering that the contrastive log-ratio upper-bound MI estimator [48] is consistent with disentanglement, we leverage it to estimate the probability log-ratio between the positive pair logp(v|u) and the negative one $\log p(v^{\prime }|u)$. But the conditional relation between *v* and *u* is unavailable in our case. Hence, we utilize a likelihood estimator *Q* to predict a variational distribution q(v|u) for approximating p(v|u). Overall, the objective function of the encoder $E_{semg}$ can be formulated as Equation (2).

(2) $$\begin{array}{c} \min_{E_{semg}}L_{inter}\left(u;v\right)=E_{\left(u,v)∼p(u,v\right)}[\log\left(q\left(v|u\right)\right]\\ -E_{\left(u,v^{\prime }\right)∼p\left(u\right)p\left(v\right)}[\log\left(q\left(v^{\prime }|u\right)\right] \end{array}$$

where *q* denotes the estimated possibility likelihood with the estimator *Q*. Meanwhile, *Q* is trained to minimize the KL divergence [49] between the true conditional probability distribution p(v|u) and the variational one q(v|u) as Equation (3).

(3) $$\min_{Q}L_{KL}\left(u,v\right)=D_{KL}\left[q\left(v|u\right)||p\left(v|u\right)\right]$$

In this paper, we assume that q(v|u) follows a Gaussian distribution, so Equation (3) can be solved via maximum likelihood estimation.

**Overall Objectives** Given the pairs of input sEMG sequences $\left(x_{i}^{t},x_{j}^{t}\right)$, the overall objective in the first stage is shown as Equation (4):

(4) $$\begin{array}{c} \min_{E}\left[\sum_{i}MI\left(x_{i}^{t};u_{i}^{t}⊙v_{i}^{t}\right)+\lambda_{1}\sum_{i\neq j}MI\left(u_{i}^{t};u_{j}^{t}\right)\right]\\ +\min_{E}\lambda_{2}\sum_{i}L_{inter}\left(u_{i}^{t};v_{i}^{t}\right)+\min_{Q}\sum_{i}L_{KL}\left(u_{i}^{t},v_{i}^{t}\right) \end{array}$$

where $u_{i}^{t}$ and $v_{i}^{t}$ denote the gesture-relevant representation and trial-relevant representation by feeding $x_{i}^{t}$ into $E_{semg}$. And $u_{j}^{t}$ and $v_{j}^{t}$ are similarly obtained by feeding $x_{j}^{t}$ into $E_{semg}$. In addition, $lambda_{1}$ and $lambda_{2}$ denote the weights of corresponding loss items.

#### 3.2.2. Stage 2: Cross-Modal Knowledge Distillation

To further enhance the discrimination of the representation learned in the first stage, we leverage cross-modal knowledge distillation to model the relationship between sEMG signals and hand movements. Specifically, we utilize a typical knowledge-distillation [41] method to map the feature spaces between these two modalities. Our target is to learn the invariant information that hand movements carry across different trials and force the sEMG encoder to mimic it. The procedure for this stage is as follows: Firstly, a teacher network (i.e., $E_{pose}\circ C_{pose}$) is trained with supervision to classify hand gestures using the modality of hand movements. Usually, the hand movements are captured using data gloves or artificially generated in accordance with the transition of a specific hand gesture. Secondly, a student network ($E_{semg}\circ C_{semg}$), which is initialized in the first stage, is trained jointly with classification loss and Kullback–Leibler (KL) divergence loss [41] to the output of the teacher network $E_{pose}\circ C_{pose}$. 

**Objectives** We denote the input of the Softmax layer in the teacher network and the student network as $Z^{\prime }=\left(z_{1}^{\prime },z_{2}^{\prime },\ldots ,z_{N}^{\prime }\right)$ and $Z=(z_{1},z_{2},\ldots ,z_{N})$, respectively. The classification loss is computed via the cross-entropy loss between predictions and ground truth as Equation (5).

(5) $$L_{CE}=-E\left[\sum_{c=1}^{N}I_{c}\left(y_{i}\right)\log\left(\frac{e^{z_{c}}}{\sum_{j}e^{z_{j}}}\right)\right]$$

where $I_{c}$ is the indicator function for $y_{i}$ equal to *c* and *N* denotes the number of gestures to be identified. On the other hand, the formulation of KL divergence loss for the two modalities (i.e., sEMG signals and hand movements) is given as Equation (6).

(6) $$L_{\mathrm{KL}}=E\left[\sum_{c=1}^{N}\left(p\left(x_{c}\right)\log p\left(x_{c}\right)-p\left(x_{c}\right)\log q\left(x_{c}\right)\right)\right]$$

In Equation (6), $p(x_{c})$ and $q(x_{c})$ are obtained by feeding $Z^{\prime }$ and *Z* into the Softmax layer, respectively. Their formulations are displayed in Equation (7):

(7) $$p\left(x_{c}\right)=\frac{e^{\frac{z_{c}^{\prime }}{T}}}{\sum_{j}e^{\frac{z_{j}^{\prime }}{T}}},\;q\left(x_{c}\right)=\frac{e^{\frac{z_{c}}{T}}}{\sum_{j}e^{\frac{z_{j}}{T}}}$$

where *T* denotes the temperature-scaling hyperparameter. It is commonly set to 1; a higher value makes the probability distribution over gesture labels softer [41]. Then, the overall loss is computed as Equation (8):

(8) $$L_{\mathrm{overall}}=\left(1-\alpha \right)L_{CE}+\alpha L_{KL}$$

where α is the balance weight of KL divergence loss.

## 4. Results

### 4.1. Datasets and Evaluation Metrics

#### 4.1.1. Datasets and Data Preprocessing

We conducted evaluations on seven sparse multichannel sEMG datasets [20,21,22,23] (denoted as NinaPro DB1-NinaPro DB7). The specific information of these seven datasets is displayed in Table 1. There are multiple trials in each NinaPro dataset, where a trial represents a repetition of performing a gesture with equipped electrodes. In some NinaPro databases (i.e., NinaPro DB1, NinaPro DB2 and NinaPro DB5), additional modalities such as acceleration and hand poses are recorded. However, hand poses are unavailable in the remaining NinaPro datasets. With regard to this situation, pseudo hand poses are generated by simulating the dynamic process of hand pose variation following [16]. Specifically, the hand pose that a hand gesture ends with is estimated at first, and then a spherical interpolation between the neutral hand pose and the estimated ending hand pose is conducted to obtain hand movements aligned with sEMG signals.

We adopt the same data-preparation procedure as the previous work [8,9,16] for a fair comparison. To mitigate noise, a low-pass Butterworth filter and an RMS filter are utilized for NinaPro DB1 and the remaining datasets, respectively. Subsequently, each trial of sEMG signals is segmented using a sliding window over 200 ms to satisfy real-time usage constraints [50] following previous work [8]. Lastly, μ-law normalization [51] is leveraged to normalize the filtered sEMG signals in terms of Equation (9).

(9) $$T\left(x_{i}^{t}\right)=sign\left(x_{i}^{t}\right)\frac{\ln(1+\mu |x_{i}^{t}|)}{\ln(1+\mu )}$$

where sign is an indicator function that equals 1 if the input is larger than 0 and otherwise is −1. And μ is set to 256 in this work.

#### 4.1.2. Evaluation Metrics

The evaluation metric in this paper is cross-trial gesture recognition accuracy, which is the same as [8,16]. Specifically, all the trials of each subject are divided into a training set and a testing set. The gesture-recognition accuracy is obtained by training our model on the training set and evaluating it on the testing set. Then, the mean gesture-recognition accuracy of all the subjects is computed as the evaluation metric. The specific split strategy of trials is described in Table 1.

### 4.2. Implementation Details

Our network *sEMGXCM* and training scheme *sEMGPoseMIM* are implemented with PyTorch, and their codes will be open-sourced online upon acceptance. In the first stage of *sEMGPoseMIM*, $E_{semg}$ is initialized using the Xavier Initialization method, and an SGD optimizer with a batch size of 128 is leveraged for all the datasets. The likelihood estimation network *Q* consists of two fully connected layers. $E_{semg}$ and *Q* are simultaneously trained, and their learning rates are initialized at 0.001 and 0.005, respectively. The training epochs of $E_{semg}$ and *Q* are both set to 30. In the second stage of *sEMGPoseMIM*, the architecture of $E_{pose}$ is derived from XceptionTime. Both $C_{semg}$ and $C_{pose}$ consist of a fully connected layer and a Softmax layer whose output dimension equals the number of gestures to be classified. An SGD optimizer with a learning rate set to 0.1 is employed and 28 training epochs are conducted while the learning rate is reduced by a factor of 0.1 at the 16th and 24th epochs.

Next, we present how to generate pairs of sEMG signals of the first stage. We need to align the trials of each subject, due to the fact that the time lengths of trials slightly vary. Note that all the trials of each gesture follow the same dynamic process, which consists of three phases, making, holding, and ending gestures; we can align the trials via their minimum length by dismissing the information of the ending phase. After that, given an anchor sEMG $x_{i}^{t}$ from trial *i*, we randomly select another trial *j* and sample from it at time step *t* to obtain the positive sEMG $x_{j}^{t}$.

### 4.3. Comparison of Networks on Cross-Trial sEMG-Based Gesture Recognition

In this part, we conduct a fair comparison between four different networks, GengNet [5], XceptionTime [9], XCM [14] and the proposed network *sEMGXCM*, on seven sparse multichannel sEMG databases (i.e., NinaPro DB1-NinaPro DB7). We train these networks from scratch on these seven datasets using the evaluation metric of cross-trial gesture recognition accuracy. As shown in the parentheses of Table 2, the proposed improved network *sEMGXCM* outperforms the other three networks. Among these networks, GengNet achieves the lowest cross-trial gesture recognition accuracy, and it exhibits the highest performance on NinaPro DB1 while demonstrating the lowest performance on NinaPro DB3. Compared with the state-of-the-art network (i.e., XceptionTime), our network *sEMGXCM* achieves significant improvements of +5.4%, +2.9%, +1.2%, +7.5%, +5.6%, +5.7% and +5.3% on NinaPro DB1-NinaPro DB7.

Note that *sEMGXCM* is derived from XCM [14]; we compare their performances to validate the superiority of *sEMGXCM* for the specific task of sEMG-based gesture recognition. Table 2 shows that *sEMGXCM* achieves higher recognition accuracies than XCM on the evaluated datasets. On the other hand, we leverage the Wilcoxon signed rank test (*p* < 0.05) on each dataset to demonstrate the significance of the improvements brought by *sEMGXCM*. And the improvements and *p*-values (in brackets) are +0.7% (0.0176), +1.3% (0.0067), +0.5% (0.0218), +0.8% (0.0149), +1.1% (0.0097), +0.3% (0.0432) and +0.3% (0.0419), respectively. Thus, we can infer that the additional self-attention layer and the modified spatial stream contributed to the significant improvements.

### 4.4. Effectiveness of sEMGPoseMIM

To demonstrate the effectiveness of the proposed training scheme *sEMGPoseMIM*, we trained four networks, GengNet [5], XceptionTime [9], XCM [14], and the improved network *sEMGXCM*, using the training scheme *sEMGPoseMIM*. The experiments were also conducted on NinaPro DB1-NinaPro DB7, and cross-trial gesture recognition was adopted as the evaluation metric. The comparisons between training from scratch and training via *sEMGPoseMIM* are displayed in Table 2. We can see that *sEMGPoseMIM* outperforms the scheme of training from scratch regardless of the network architectures. The improvements achieved by training GengNet using *sEMGPoseMIM* are +1.1%, +9.2%, +16.0%, +2.6%, +4.9%, +3.7%, and +3.2%. As the performance of GengNet is far from optimal, the improvements brought about by *sEMGPoseMIM* are much more significant compared with the other three networks. With regard to the other three networks, *sEMGPoseMIM* could achieve improvements of at least +1.2% on the evaluated datasets. These results indicate the significant effectiveness of the proposed training scheme *sEMGPoseMIM*.

Furthermore, we compared the performance of training *sEMGXCM* using the training scheme *sEMGPoseMIM* with that of existing sEMG-based gesture recognition approaches. This comparison was also conducted on NinaPro DB1-NinaPro DB7, and the evaluation metric of cross-trial gesture recognition accuracy was adopted. As shown in Table 3, our method (i.e., *sEMGXCM*+*sEMGPoseMIM*) outperformed the state-of-the-art approach CMAM [16], where hand poses were directly generated using sEMG signals and then fused with the input sEMG. The specific improvements achieved using our method on NinaPro DB1-NinaPro DB7 were +1.3%, +1.5%, +0.8%, +2.6%, +1.7%, +0.8%, and +0.6%, which provides further evidence of the effectiveness of *sEMGPoseMIM*.

### 4.5. Variation on Each Stage

We also validated the effects of each stage in *sEMGPoseMIM* by comparing four training schemes, training from scratch, only on the first stage, only on the second stage, and on both stages (i.e., *sEMGPoseMIM*). To train *sEMGXCM* only on the first stage, we fine-tuned the *sEMGXCM*, whose parameters are initialized in the first stage via cross-trial mutual information maximization. With regard to the second stage, we initialized the sEMG encoder using the Xavier Initialization method and trained the *sEMGXCM*, as in the second stage.

As shown in Table 4, each stage of *sEMGPoseMIM* contributes to its performance improvement. Compared with training from scratch, the cross-trial mutual information maximization in the first stage brought improvements of +0.7%, +0.2%, +2.1%, +0.7%, +0.8%, +0.8% and +0.7% over NinaPro DB1-NinaPro DB7. The effects of cross-modal knowledge distillation in the second stage over NinaPro DB1-NinaPro DB7 are +0.5%, +0.8%, +1.7%, −0.9%, +0.6%, 0.0% and +0.2%. On most datasets except NinaPro DB4 and NinaPro DB6, training only in the second stage outperformed training from scratch. When both stages were utilized, the performance improvements over the evaluated datasets were significant and improvements of at least +1.0% were achieved. These experimental results indicate that both stages of *sEMGPoseMIM* are essential for enhancing the classification model.

## 5. Discussion

In the proposed network *sEMGXCM*, we used the GengNet architecture to extract spatial features. The reason why we chose it lies in a good trade-off between the number of parameters and performances on the NinaPro datasets. In addition, GengNet achieved superb performance on high-density sEMG-based gesture recognition [5], indicating that GengNet extracts more discriminative spatial features.

Furthermore, we compared the results of classic models on NinaPro DB1 and NinaPro DB2 in previous works with the experimental results of training four networks to gain a more comprehensive insight. As depicted in [20], Random Forests was adopted to train on the NinaPro DB1 and NinaPro DB2, and then recognition accuracies of 75.32% and 75.27% were obtained, respectively. We can see that *sEMGXCM* largely outperforms classic models, which further indicates the effectiveness of the proposed network.

## 6. Conclusions

In this paper, we propose a novel end-to-end convolutional neural network for cross-trial gesture recognition based on sparse sEMG signals, namely *sEMGXCM*. By capturing the spatial and temporal information of sEMG signals, as well as the correlation across different electrodes (i.e., channels), *sEMGXCM* achieves superior performances on seven sparse sEMG datasets (i.e., NinaPro DB1-NinaPro DB7). Additionally, we introduced a novel two-stage training scheme called *sEMGPoseMIM* to enhance the classification model. Specifically, a trial-invariant representation is learned using mutual information maximization in the first stage. Subsequently, the inherent relation between the sEMG signals and hand movements is modeled via cross-modal knowledge distillation to obtain a more discriminative representation. To the best of our knowledge, mutual information and cross-modal knowledge distillation are for the first time simultaneously employed for sEMG-based gesture recognition. Moreover, our training scheme *sEMGPoseMIM* is network-agnostic and can be applied to most convolutional networks for gesture recognition based on sEMG.

To validate the effectiveness of *sEMGXCM* and *sEMGPoseMIM*, we conducted comprehensive experiments on NinaPro DB1-NinaPro DB7. The comparison between *sEMGXCM* and existing networks for sEMG-based gesture recognition was performed by training these networks from scratch on the seven datasets. The experimental results show that *sEMGXCM* outperforms the state-of-the-art network for cross-trial gesture recognition based on sparse sEMG signals. Furthermore, the proposed training scheme *sEMGPoseMIM* is utilized to train four different networks (i.e., GengNet, XceptionTime, XCM and *sEMGXCM*) for validating the effectiveness of *sEMGPoseMIM*. The results demonstrate that *sEMGPoseMIM* can bring improvement for cross-trial gesture recognition based on sEMG. Furthermore, an ablation study on the effect of each stage in *sEMGPoseMIM* was conducted, and the results suggest that every stage is required, as skipping any stage leads to reduced performance.

Our future work will focus on the extension of the proposed training scheme on inter-subject or inter-session sEMG-based gesture recognition, which is much more difficult than cross-trial sEMG-based gesture recognition. We also plan to leverage a more effective approach to model the relationship between sEMG signals and hand movements, such as causal representation learning [53] and contrastive learning [54]. Furthermore, our method may lack resilience [55] because the sEMG signals are sensitive to the electrodes. However, it is truly important for human–computer interfaces to retain the resilience. We will also pay more attention to it in our future work.

## Figures and Tables

**Table 1:** Specifications of the Evaluated NinaPro Datasets. The link to the NinaPro data repositories is https://ninapro.hevs.ch/ (accessed on 9 February 2022).

Dataset	Labels	Subjects	Trials	Hand Kinematic	Channels	Sampling Rate	Training Trials	Test Trials
Ninapro DB1 [20]	52	27	10	w	10	100 Hz	1, 3, 4, 6, 7, 8, 9	2,5,10
Ninapro DB2 [20]	50	40	6	w	12	2000 Hz	1, 3, 4, 6	2, 5
Ninapro DB3 [20]	50	11	6	w/o	12	2000 Hz	1, 3, 4, 6	2, 5
Ninapro DB4 [21]	53	10	6	w/o	12	2000 Hz	1, 3, 4, 6	2, 5
Ninapro DB5 [21]	53	10	6	w	16	200 Hz	1, 3, 4, 6	2, 5
Ninapro DB6 [22]	7	10	10	w/o	16	2000 Hz	1, 3, 5, 7, 9	2, 4, 6, 8, 10
Ninapro DB7 [23]	41	22	6	w/o	12	2000 Hz	1,3,4,6	2, 5

**Table 2:** Gesture-recognition performance of the four networks through training from scratch (shown in parentheses) and training using *sEMGPoseMIM* on NinaPro DB1-NinaPro DB7. The bold entries indicate the best performance on the corresponding dataset.

Backbone	NinaPro DB1	NinaPro DB2	NinaPro DB3	NinaPro DB4	NinaPro DB5	NinaPro DB6	NinaPro DB7
GengNet [5]	78.9% (77.8%)	59.4% (50.2%)	57.0% (41.0%)	67.4% (64.8%)	78.9% (74.0%)	60.1% (56.4%)	77.8% (74.6%)
XceptionTime [9]	85.0% (83.6%)	83.4% (82.1%)	55.0% (53.0%)	71.7% (70.2%)	89.0% (86.7%)	61.3% (59.5%)	86.5% (84.1%)
XCM [14]	90.5% (88.3%)	84.8% (83.7%)	65.0% (63.7%)	78.1% (77.4%)	94.0% (92.0%)	66.4% (64.9%)	90.5% (89.1%)
sEMGXCM	**91.4% (89.0%)**	**86.3% (85.0%)**	**66.5% (64.2%)**	**78.7% (77.7%)**	**94.2% (92.3%)**	**66.9% (65.2%)**	**91.2% (89.4%)**

**Table 3:** Gesture-recognition accuracies (%) on the benchmark NinaPro sEMG databases. The reported performance was achieved with sEMG windows of 200 ms. The bold entries indicate the best performance on the corresponding dataset.

	NinaPro DB1	NinaPro DB2	NinaPro DB3	NinaPro DB4	NinaPro DB5	NinaPro DB6	NinaPro DB7
GengNet [5]	77.8%	50.2%	41.0%	64.8%	74.0%	56.4%	74.6%
DuNet [52]	79.4%	52.6%	41.3%	64.8%	77.9%	56.8%	74.2%
HuNet [11]	87.0%	82.2%	46.7%	68.6%	81.8%	58.0%	80.7%
WeiNet [8]	88.2%	83.7%	64.3%	51.6%	90.0%	64.1%	88.3%
CMAM [16]	90.1%	84.8%	65.7%	76.1%	92.5%	66.1%	90.6%
Our Method	**91.4%**	**86.3%**	**66.5%**	**78.7%**	**94.2%**	**66.9%**	**91.2%**

**Table 4:** Effects of each stage on gesture-recognition performance over NinaPro databases. The baseline method in this table refers to directly training *sEMGXCM* from scratch. The bold entries indicate the best performance on the corresponding dataset.

	NinaPro DB1	NinaPro DB2	NinaPro DB3	NinaPro DB4	NinaPro DB5	NinaPro DB6	NinaPro DB7
From Scratch	89.0%	85.0%	64.2%	77.7%	92.3%	65.2%	89.4%
Stage 1 Only	89.7%	85.2%	66.3%	78.4%	93.1%	66.0%	90.1%
Stage 2 Only	89.5%	85.8%	65.9%	76.8%	92.9%	65.2%	89.6%
Two Stages	**91.4%**	**86.3%**	**66.5%**	**78.7%**	**94.2%**	**66.9%**	**91.2%**

## Data Availability

The data presented in this study are available on request from the corresponding author.
